# Consider Carcinoma Cuniculatum in Recurrent Foot Ulcer: A Case Report and Literature Review

**DOI:** 10.1055/a-2316-3824

**Published:** 2024-06-10

**Authors:** Francisco Nunes-Abreu, Ruben Hidalgo-Caro, Elena Lorda-Barraguer, Victor Cristóbal-Redondo, F. Javier Céspedes-Guirao

**Affiliations:** 1 Department of Plastic Surgery, Hospital General Universitario Dr. Balmis, Alicante, Spain; 2 Department of Anatomopathology, Hospital General Universitario Dr. Balmis, Alicante, Spain

**Keywords:** carcinoma cuniculatum, foot reconstruction, through-and-through defect, gracilis free flap

## Abstract

Carcinoma cuniculatum is a variant of squamous cell carcinoma, characterized by a slow growth with progressive crypt-like invasion of deep tissue. This tumor is frequently misdiagnosed as a benign skin lesion both clinically and histopathologically. The final diagnosis is often delayed as it requires a large sample biopsy. We report the case of a 67-year-old patient who presented to us with a recurring chronic ulcer over a surgical scar of 5 years of evolution.

Only after a wide resection of the chronic ulcer was it possible to achieve the correct diagnosis of this large and poorly evolving carcinoma. The subsequent reconstruction with a musculocutaneous gracilis free flap allowed the patient to walk again.

## Introduction

Carcinoma cuniculatum is a rare form of low-grade squamous cell carcinoma, occurring mainly on the sole of the foot. 1 It usually presents as an exophytic lesion, exuding foul-smelling material from numerous sinuses. Histologically, it is characterized by well-differentiated keratinocytes with crypt-like invasion of the deep tissue. The tumor manifests a slow growth with progressive invasion of surrounding tissues, although it has limited metastatic ability. Its evolution can reach the destruction of deep structures such as bone. 2 This tumor is frequently misdiagnosed as other benign skin lesions such as warts, and for this reason, recurrence and invasion of underlying tissue are commonly reported in the literature at the time of diagnosis. Given these characteristics and invasion of local tissue, an ample excisional biopsy is required to allow for the correct diagnosis. 3

We report a case of plantar carcinoma cuniculatum that required ample resection which included disarticulation of the third and fourth toes at Lisfranc joint. The use of a free musculocutaneous gracilis flap was required to cover the vast transfixing defect in the central part of the foot.

## Case

A 67-year-old female with an ulcer in the left foot presented for treatment with a medical history of seropositive rheumatoid arthritis. During the 20 years of evolution of her disease she received numerous biological systemic treatments, achieving a state of chronic immunosuppression. The beginning of this ulcer were several hyperkeratotic lesions in the left foot over the scar of a rheumatoid foot correction surgery several years ago, this ulcer produced great pain and prevented her from walking. Despite six previous attempts to treat this ulcer, it kept recurring in the form of a whitish lesion, with grooves, cracks, and irregularities reminiscent of a cauliflower, appearing on the edges of surgical scars. This hyperkeratotic lesions rapidly evolved into an ulcer that was accompanied by abscesses in the soft tissues. After 5 years of evolution, the patient was finally referred to our department for treatment of the chronic ulcer.

**Fig. 1:**
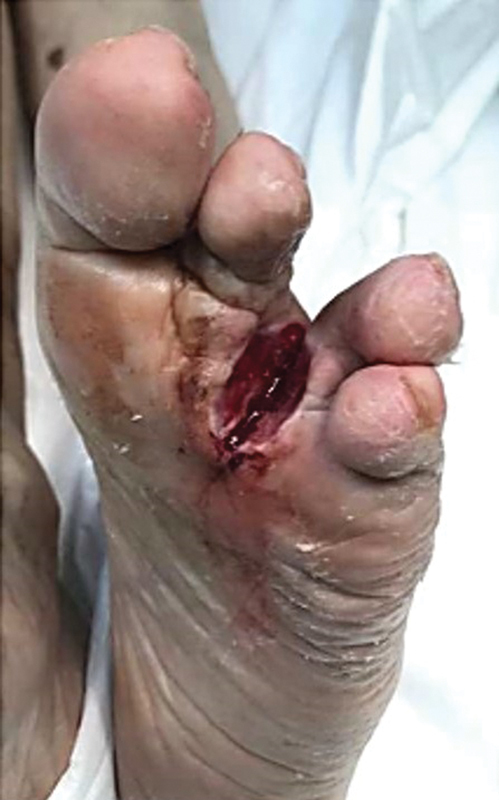
Preoperative image of left foot presenting a recurrent ulcer over a surgical scar.

**Fig. 2:**
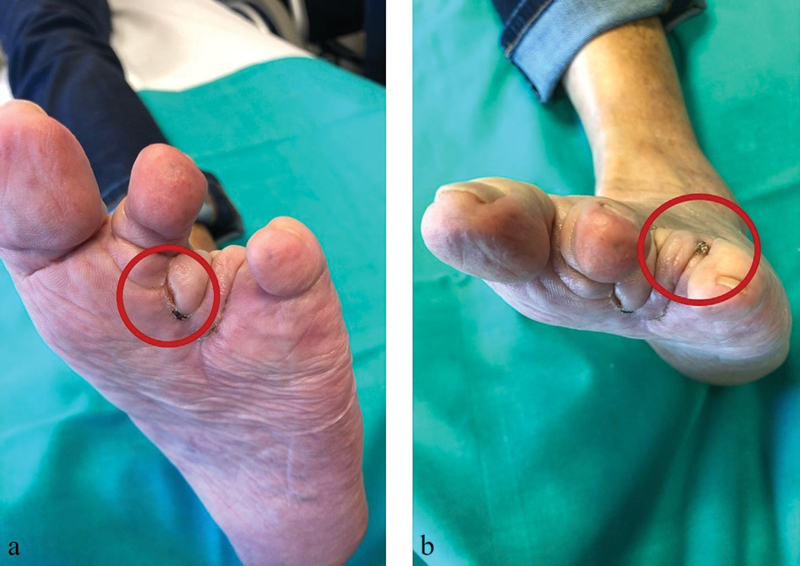
Two-month follow-up after debridement of necrotic tissue and coverage with a local flap. Early recurrence of the hyperkeratotic lesions can be visualized within the red circles.

Given the poor evolution of the ulcer and previous biopsy findings of benign hyperkeratotic proliferation, compatible with a plantar wart, a surgery was performed for resection of the remaining devitalized tissue ( Fig. 1 ). This comprised amputation of the fourth toe and reconstruction with a local fillet flap. The initial evolution was good and pathological report showed no relevant findings. After 8 months of follow-up, a new hyperkeratotic lesion (5 mm in diameter) appeared over the surgical scar. This hyperkeratotic lesion was biopsied and described, once again, as common wart which was treated with cryotherapy. After 2 months, the lesion recurred ( Fig. 2 ) presenting with an abscess of the plantar foot. An MRI was performed confirming osteomyelitis with multiple fistulous tracts.

**Fig. 3:**
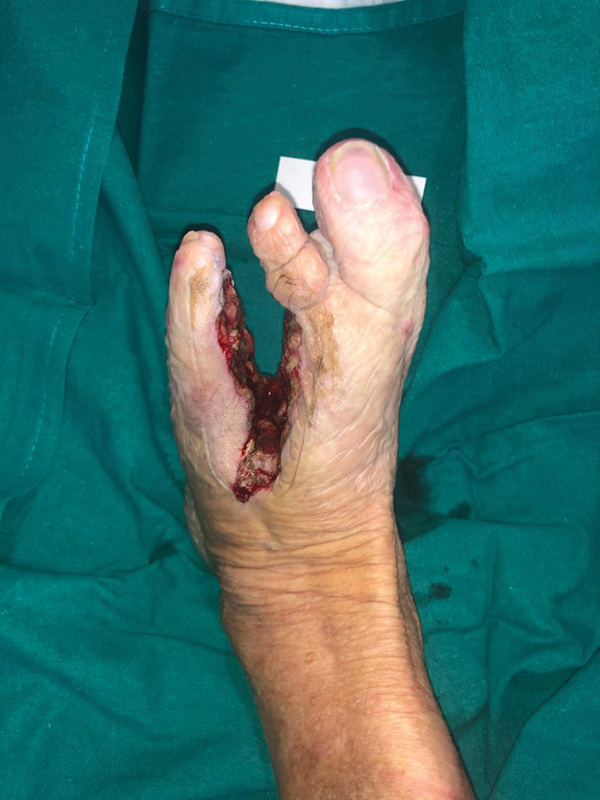
Resulting defect after wide resection including third and fourth metatarsal and overlying soft tissue.

**Fig. 4:**
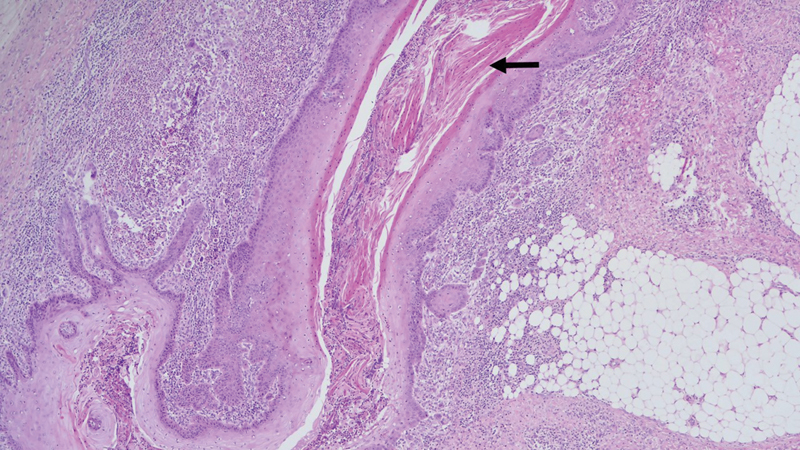
Pathological image of the tumor showing the sinus tracts with keratin in its interior (arrow). H&E at ×4 magnification.

**Fig. 5:**
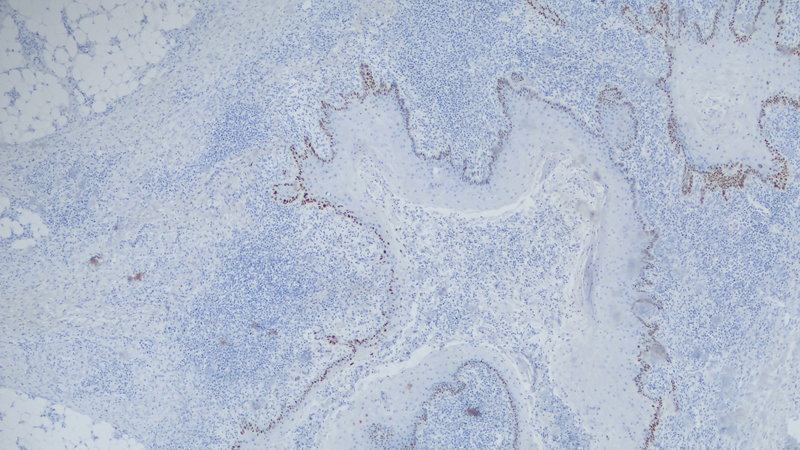
Pathological image of the tumor with immunohistochemical staining of p53, showing a positive result represented by the tinted blue nuclei. Magnified ×20.

Given the inadequate evolution of the case, an infiltrative malign skin lesion was suspected and it was decided to proceed with an ample resection of the affected tissue around the ulcer (leaving a defect of about one-third of the total forefoot volume). This included a disarticulation of the third and fourth toes at the level of Lisfranc joint ( Fig. 3 ). A vacuum-assisted therapy was used as the bridge treatment waiting for the result of the pathology study. The pathologist diagnosed the lesion as a squamous cell carcinoma subtype cuniculatum, describing proliferation of well-differentiated squamous epithelium organized in deep irregular nests that penetrate deep into the soft tissue and bone ( Fig. 4 ). To aid with the histological diagnosis, both human papilloma virus (HPV) polymerase chain reaction (PCR) with serotypes and immunohistochemical staining of p53 were employed, the former was negative and the latter was positive which proved helpful in assisting in the definitive diagnosis ( Fig. 5 ). The surgical margins were confirmed free from tumor.

**Fig. 6:**
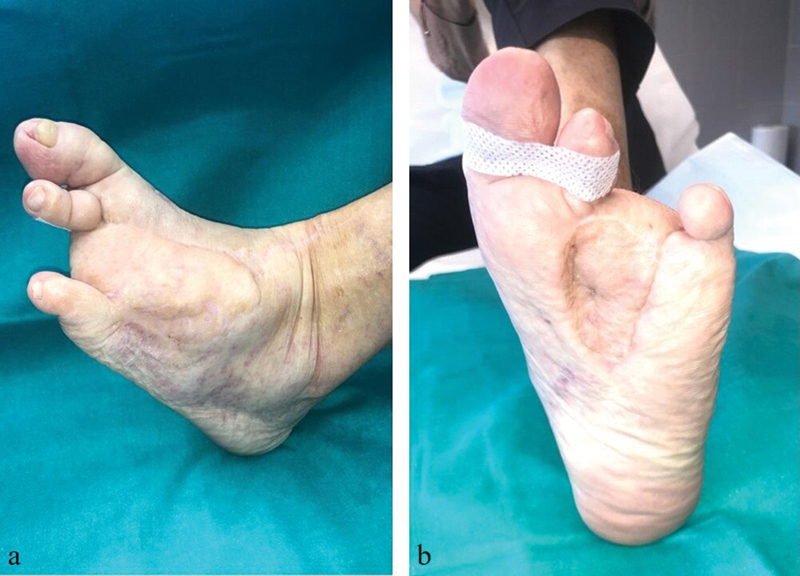
Follow-up 6 months after surgery showing adequate coverage with gracilis myocutaneous free flap in both dorsal and plantar sides.

After confirmation of free margins, a microsurgical reconstruction with a gracilis myocutaneous free flap was planned. Standard microvascular anastomosis was performed to the dorsalis pedis artery and the flap was inset in a sandwich manner providing skin coverage for both dorsal and plantar foot, the muscle was used to occlude the death space between the second and fifth toes ( Fig. 6 ). The evolution of the patient was favorable and walking was restarted 6 months after surgery; after 48 months of follow-up, there were no signs of recurrence.

## Discussion

The carcinoma cuniculatum, also known as epithelioma cuniculatum, was originally described by Aird et al in 1954 as rare subtype of squamous cell carcinoma which presented tunnel-like structures that penetrated underlying tissue and affected the sole of the feet. 4 Although it typically affects the foot, reports have been made of this tumor appearing in other areas, such as the leg, hand, or the gluteal region. 5 6 7

The etiology of the carcinoma cuniculatum is not clear, the factors associated with the appearance of this tumor are usually recurrent traumatisms, burns, and chronic inflammation related to the presence of ulcers in the lower limb. 6 Some authors advocate for a relation with HPV type 6 and 11, multiple reports show positive HPV findings, but this association has not yet been clearly established. 8 In our case report, HPV PCR and serotypes were studied but results were negative.

The hallmark of this tumor is its initial benign appearance both clinically and pathologically, paired with the capacity to recur and infiltrate underlying tissue. The tumor usually starts as a seemingly benign skin lesion that is either left untreated or it is incompletely removed. Given the characteristic depth of its sinuses' tracts and the lack of atypia, if the initial biopsy is not deep enough the pathologist commonly reports hyperkeratotic proliferation that can be compatible with a common wart or other benign lesions. 1 2 5 6 7 Following the incomplete resection, the tumor will recur, and if left untreated it will eventually ulcerate while slowly growing and invading underlying soft and bone tissue, sometime mimicking an osteomyelitis. 9 10 Despite the potential invasion of underlying deep tissue, distant metastasis is extremely rare, only five cases have been reported. 11

Recurrence and late diagnosis are frequently reported in the literature, some case series report an average of 5 years of evolution before correct diagnosis, at this time the tumors average 4 cm in size and more than 50% of the patients present chronic ulceration within the tumor. 5 6 It is also not uncommon for the tumor to have been biopsied previous to definitive diagnosis and reported as benign findings such as a wart or reactive epidermal hyperplasia. 1 2 5 6 7 10 11 12 For these reasons, it is important to think of carcinoma cuniculatum when presented with a repeating ulcer on the foot, arising from a seemingly benign lesion that keeps recurring despite attempts of surgical removal.

Besides viral warts, some of the other conditions that have been reported to be mimicked by this tumor and thus should be included in the differential diagnosis are keratoacanthoma, giant seborrheic keratosis, giant condyloma acuminatum, epidermic cyst, pyogenic granuloma, and amelanocytic melanoma among others. 5 6 7 10 11 In the later stages, if it evolves into a chronic lesion with aggressive invasion of underlying tissue the differential diagnosis should also include Marjolin's ulcer. Additionally, some authors have emphasized the importance of differentiating between carcinoma cuniculatum and verrucous skin lesions on the foot in diabetic neuropathy, since it is not uncommon for the demography of both diseases to overlap and differential diagnosis can be difficult if tissue biopsy is not taken appropriately. 12 Histological diagnosis of this tumor can also pose challenges due to its similarity to other lesions. For this reason, diagnostic criteria for this tumor have been proposed, the main pathological findings in this tumor are squamous proliferations without atypia of the keratinocytes, sinuses, and crypts deep to the skin surface lined with epithelium, these crypts contain laminated keratin . 2 Nakamura et al have discussed the role of mutated p53 as a tool to aid differentiation between this tumor and the verrucous lesions of the diabetic foot. 13 In our case report, a p53 strain was employed by the pathologist which helped achieve the final diagnosis.

There have also been multiple publications about the use of imaging studies such as MRI or CT scan to help identify the fistulous tract of the tumor and its invasion of deep tissue, Wasserman et al describe a correlation between the MRI images and the pathological findings in their cases. 14 15 In our case, the MRI scan showed signs of osteomyelitis and abundant fistulous tracts which helped us decide towards a radical resection of affected tissue.

The recommended treatment of epithelioma cuniculatum is surgical excision, with complete tumor extirpation and 5 mm margins. Small resections normally recur which can be a source of greater morbidity in later interventions. 3 6 Some cases have been reported using Mohs surgery to treat these lesions, a recently published systematic review of treatment options showed Mohs surgery as a promising tool but not superior to conventional surgery. Nonsurgical treatments, such as imiquimod, have also been proposed in certain cases but they have not shown superiority to surgical treatment. 3

Given the scarce amount of tissue in the foot, the reconstruction following the resection of malignant skin lesions commonly requires the use of local flaps or skin grafts. In the context of this tumor, the use of advanced reconstructive techniques, such as free flaps, has only been reported two times, both describe the use of a radial forearm flap to cover sizeable defects in the foot. 16 17 The defect we presented in this report was a large transfixing defect that posed greater complexity than the ones described in the previously cited articles. In fact, to our knowledge, there are only four publications in the whole literature regarding the reconstruction of through-and-through defects of the foot similar to the one we presented. 18 19 20 In these publications, the authors describe a total of nine cases of transfixing defects caused by gunshot wounds, in all the cases the reconstructions is carried out with a musculocutaneous free flap, the most commonly used flap being the gracilis. 18 19 20 The tridimensional reconstruction that we report is bigger than most of the ones found in the literature. We agree with other authors that the gracilis myocutaneous flap provides sufficiently bulky, pliable, and well-irrigated tissue that allows to cover both sides of the foot; furthermore, the bulk of the muscles occludes the dead space and allows weight-bearing and walking without the need for osseous reconstruction. Additionally, this flap has minimal donor site morbidity in terms of function and cosmesis with hidden medial thigh scar.

In conclusion, recurring and chronic ulcers in the foot should always alert our attention to discard secondary tissue malignancies. Carcinoma cuniculatum is an infrequent tumor that has been described with a tendency to recur and to be easily confused both clinically and pathologically with other more benign skin lesions. This tumor should be taken into account when making a differential diagnosis in chronic ulcers or recurring lesions in the foot. An aggressive resection of deep tissue is required to both confirm diagnosis and prevent recurrence.
